# A Study on the Application of Near Infrared Hyperspectral Chemical Imaging for Monitoring Moisture Content and Water Activity in Low Moisture Systems

**DOI:** 10.3390/molecules20022611

**Published:** 2015-02-03

**Authors:** Eva Achata, Carlos Esquerre, Colm O’Donnell, Aoife Gowen

**Affiliations:** School of Biosystems Engineering, University College Dublin, Dublin 4, Ireland; E-Mails: eachata@gmail.com (E.A.); carlos.esquerre@ucd.ie (C.E.); colm.odonnell@ucd.ie (C.O.)

**Keywords:** hyperspectral, near infrared, moisture content, water activity, food

## Abstract

Moisture content and water activity are key parameters in predicting the stability of low moisture content products. However, conventional methods for moisture content and water activity determination (e.g., loss on drying method, ‎Karl Fischer titration, dew point method) are time consuming, demand specialized equipment and are not amenable to online processing. For this reason they are typically applied at-line on a limited number of samples. Near infrared hyperspectral chemical imaging is an emerging technique for spatially characterising the spectral properties of samples. Due to the fast acquisition of chemical images, many samples can be evaluated simultaneously, thus providing the potential for online evaluation of samples during processing. In this study, the potential of NIR chemical imaging for predicting the moisture content and water activity of a selection of low moisture content food systems is evaluated.

## 1. Introduction

Water activity (aw), and moisture content (MC) are the most valuable characteristics for assessing the quality and stability of dried foods [1]. Moisture content represents a measure of the quantity of water in a product, providing information about yield, quantity and texture, but does not provide reliable information about microbial safety. Water activity represents a measure of water that is available to react with other molecules and participate in spoilage reactions such as enzymatic browning or microbial growth. Thus, aw is an indicator of stability with respect to microbial growth, biochemical reaction rates and physical properties. The relationship between moisture content and water activity (aw) is complex. An increase in aw is almost always accompanied by an increase in the moisture content, but in a nonlinear trend, called the moisture sorption isotherm, at a given temperature. Water activity and moisture content are complementary and together provide a complete moisture analysis of a given system. 

The aw of low-moisture foods is dependent on storage relative humidity and temperature. Although microbial spoilage is prevented at aw below 0.60, low-moisture foods are prone to gain moisture which can be followed by undesirable changes, such as structural transformations, enzymatic changes, browning and oxidation, depending on aw and temperature. For example, in wafers, texture and mechanical characteristics critically depend on moisture content, due to the plasticization effect in the starch matrix [2]. If a wafer after baking is too dry, then it is brittle and breaks easily, making further processing difficult, but if MC is too high, the texture is affected and bacterial growth may result in a significant decrease of the product shelf-life [2]. In instant coffee powder high MC interferes with the flowing characteristics and agglomeration of the product, while over drying can result in a loss of volatile compounds affecting flavour. In pulse foods, such as soybean kernels, high MC is linked with mould growth, while low MC results in damage of skin and affects hydration properties [3].

Traditional techniques for determination of MC and aw (e.g., loss on drying method, ‎Karl Fischer titration, dew point method) are time consuming, demand specialized equipment and are not amenable to online processing. Near Infrared (NIR) spectroscopy has become a widely used method in food analysis because it is a fast method and requires little or no sample preparation [4]. For these reasons it has become one of the most efficient tools for on/at-line control and monitoring of processes in food [5,6]. NIR spectroscopy is well suited for MC determination because water shows strong absorption bands in the NIR. Liquid water exhibits three major vibrational absorbance peaks in IR due to asymmetric stretching, symmetric stretching and bending vibrations (v_as_, v_s_ and v_ds_ respectively) of the covalent OH bond in the water molecule, located at approximately 2734, 6270 and 2662 nm respectively. Overtones of these fundamental frequencies occur in the NIR and can be estimated using the approach suggested by Weyer and Workman [7]. They proposed a rule of thumb for estimation of the overtones of fundamental IR vibrations, where the overtones can be estimated to occur within a range of values as shown in Equation (1) where λ represents the wavelength in nanometres (nm), the subscripts 1 and 2 refer to fundamental and 1st overtone.

(1) $$\lambda_{2}=(\frac{\lambda_{1}}{2},\frac{\lambda_{1}}{2}+\lambda_{1}(0.05))$$

Using Equation (1) the range of expected wavelength positions for possible overtones and combination bands of the fundamental water absorption bands can be calculated (Table 1).

**Table 1:** Estimated positions of overtones and combinations of fundamental asymmetric, symmetric and bending vibrations of the water molecule (v_as_, v_s_ and v_ds_ respectively) calculated using Equation (1).

Assignment	Estimated Position (nm)
2v_as_	1367–1504
2v_ds_	3135–3448
2v_s_	1331–1464
3v_as_	911–1048
3v_ds_	2090–2403
3v_s_	887–1020
v_as_ + 2v_s_	895–954
2v_as_ + v_s_	903–961
v_ds_ + 2_vs_	1098–1187
v_as_ + v_ds_ + v_s_	1110
2v_as_ + v_ds_	1112–1213
v_as_ + v_s_	1349
2v_ds_ + v_s_	1440–1502
v_as_ + 2v_ds_	1461–1525

There are many applications of NIR for the determination of MC but there are very few works which explore the use of single point NIR spectroscopy for prediction and understanding of aw in food systems [8,9,10,11]. In many cases food systems are not homogenous, therefore the spectrum collected will depend of the position on the sample where is it acquired. A method which provides spatial and spectral information of such food systems would therefore be desirable.

Hyperspectral imaging (HSI) is an emerging platform technology that integrates conventional imaging and spectroscopy to attain both spatial and spectral information from an object; it is a powerful process analytical tool for non-destructive food analysis, food safety and quality assessment, for contaminant detection, for defect identification, constituent analysis and quality evaluation. Hyperspectral images, known as hypercubes, can be represented as three-dimensional blocks of data, comprising of two spatial and one wavelength dimension [12]. Each pixel in a hyperspectral image contains a spectrum representing the light absorbing and/or scattering properties of the spatial region represented by that pixel. The resulting spectrum can be used to estimate chemical composition or physical properties of the spatial region represented by that particular pixel. Compared with traditional NIR spectroscopy, HSI has the advantage that it can provide spatial information, which is useful for assessment of sample homogeneity and identification of local defects or contamination. 

The objective of this research was to investigate the application of near infrared hyperspectral imaging and chemometrics for prediction of behaviour of moisture content and aw in different low moisture food systems.

## 2. Results and Discussion

**Figure 1:**
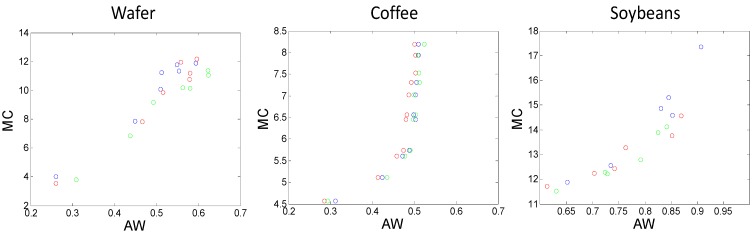
Moisture content (MC) plotted as a function of water activity (aw) for each food sample studied. Different colours represent independent replicates.

The relationship between water activity (aw) and moisture content (MC) for each product studied is shown in Figure 1. It is clear that each product demonstrates a specific relationship between aw and MC, reflecting the differences in water binding by each specific matrix. For the wafer and soybean samples the relationship is quazi-linear whereas for the coffee samples the relationship is highly non-linear: after an aw of 0.5 further increases in MC did not appreciably change the aw. The general trends observed were consistent over independent experimental replicates on different days.

**Figure 2:**
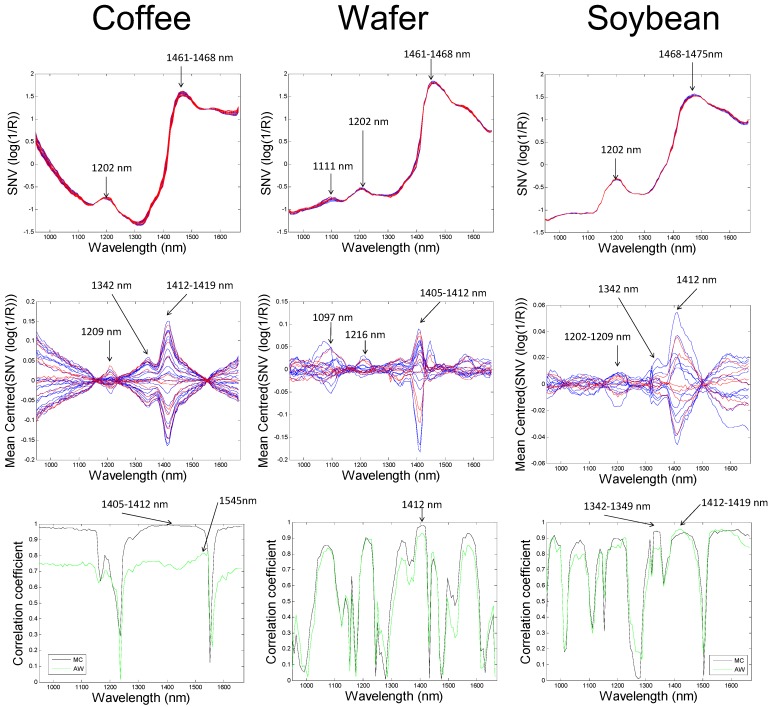
Top row: Standard normal variate (SNV(log(1/R))) pre-treated mean spectra; middle row: Mean centred SNV spectra (obtained by subtracting the mean SNV(log(1/R) spectrum from each group) of each food sample studied. Blue spectra represent calibration set and red spectra represent independent test set. Bottom row: Absolute value of correlation between SNV(log(1/R)) at each wavelength and moisture content (MC, black line) or water activity (aw, green line).

The standard normal variate (SNV) pre-treated absorption (log (1/R)) spectra of each product (coffee, wafer or soybean), including the wavelength positions of major peaks, are shown in Figure 2. The spectral profile for each product is different, due to the different molecular composition of each sample type. However, there were some common absorption peaks for each sample. For example, the major peak exhibited by the coffee and wafer samples occurred at 1461–1468 nm, while that for soybean occurred in a slightly higher wavelength range, 1468–1475. Based on Table 1, this peak can be attributed to the 1st overtones of v_as_ and v_s_ and the combination vibrations 2v_ds_ + v_s_ and v_as_ + 2v_ds_. Each product also exhibited an absorption feature at 1202 nm, which is probably related to the second overtone of the C-H stretching vibration, but may arise due to the 2v_as_ + v_ds_ water combination vibration (Table 1). In addition, a weak absorption feature at 1111 nm was observed in the spectra of the wafer samples, which may be due to the v_ds_ + 2v_s_ and v_as_ + v_ds_ + v_s_ water combination vibrations.

The mean-centred spectra of all three products provide a simple way to examine at which wavelength regions changes in absorbance due to hydration occurred (Figure 2). The samples exhibited peaks around 1202–1216, 1342 and 1402–1419 nm due to first overtone vibrations of v_as_ and v_s_*.* The latter absorption band has been previously attributed to weakly hydrogen bonded or “free” water [13], which increases in concentration as dry samples are hydrated. This water is free to participate in deteriorative reactions such as microbial growth and enzymatic browning, and thus reduces the shelf life of dry food products.

In order to further examine which wavelengths were most correlated to changes in MC and aw, a simple correlation analysis was carried out. The correlation coefficient between SNV(log(1/R)) absorbance at each wavelength and MC or aw was calculated and is plotted in the bottom row of Figure 2. It is evident that the relationship between light absorbance and hydration is different for each product. However, for each individual product the correlation between absorbance and MC was very similar to that between absorbance and aw. This can be expected due to the correlation between aw and MC previously mentioned (Figure 2). For coffee samples, the highest correlation for MC was around 1405–1412, while the highest correlation for aw occurred at 1545 nm. High correlations (*r* > 0.95) for MC were also found in the ranges 950–1146; 1314–1531 and 1580–1664 nm. Similarly, relatively high correlations (*r* > 0.7) were found in the same regions for aw. For wafer, the highest correlation between MC or aw was found at 1412 nm. High correlation coefficients (*r* > 0.85) were also found in the regions around 1076, 1209, 1342 and 1573). For soybean samples, the highest correlation for MC was at around 1342–1349 nm, while for aw the highest correlation was at 1412–1419 nm. High correlation coefficients (*r* > 0.85) were also found in the regions around 978, 1062, 1202 and 1573. This indicates that absorbance at many wavelengths in the NIR, most of which appear in the ranges shown in Table 1, are influenced by hydration.

Predictive model performance is summarised in Table 2. In general, the ability of the developed PLS models to predict the moisture content for coffee samples was better than for wafer and soybean samples, while the prediction of water activity for wafer and soybean were better than for coffee samples. This behaviour could be explained by the nonlinear relationship between MC and aw for these samples. For example after an aw of 0.5 for coffee samples the moisture content continues to change at a much faster rate than the water activity.

**Table 2:** Prediction of moisture content and aw using different pretreatments.

Product	Parameter	Sample Pretreatment Spectra	Latent Variables	RMSECV	RMSEP	R^2^
Coffee	MC	Raw	3	0.2	0.3	0.99
		SNV	6	0.1	0.3	0.99
		MSC	2	0.1	0.1	0.99
		1st derivative	1	0.2	0.2	0.99
		2nd derivative	1	0.1	0.1	0.99
	aw	Raw	2	0.045	0.042	0.57
		SNV	7	0.022	0.038	0.72
		MSC	2	0.044	0.046	0.54
		1st derivative	1	0.044	0.043	0.55
		2nd derivative	5	0.035	0.053	0.49
Wafer	MC	Raw	3	0.6	0.6	0.96
		SNV	3	0.4	0.4	0.98
		MSC	3	0.4	0.4	0.98
		1st derivative	5	0.3	0.7	0.95
		2nd derivative	5	0.4	0.7	0.95
	aw	Raw	3	0.026	0.058	0.97
		SNV	1	0.025	0.067	0.97
		MSC	1	0.025	0.067	0.97
		1st derivative	2	0.027	0.045	0.96
		2nd derivative	1	0.025	0.048	0.94
Soybeans	MC	Raw	1	0.6	0.3	0.95
		SNV	5	0.4	0.8	0.92
		MSC	5	0.4	0.8	0.92
		1st derivative	2	0.5	0.3	0.92
		2nd derivative	2	0.5	0.4	0.91
	aw	Raw	3	0.035	0.031	0.89
		SNV	1	0.036	0.029	0.91
		MSC	1	0.036	0.029	0.91
		1st derivative	1	0.035	0.031	0.87
		2nd derivative	1	0.034	0.036	0.86

Considering the coffee samples, MC was well predicted using the MSC or 2nd derivative pretreatments (*R*^2^ > 0.99 with 2 and 1 LV respectively). In contrast, the best model for predicting aw employed the SNV pretreatment and required 7 LV (*R*^2^ = 0.72). This high number of LV was required due to the highly nonlinear behaviour of water activity, as mentioned above. The best models for predicting MC of wafer samples were MSC or SNV pretreated data with 3 LV (*R*^2^ > 0.98), while for predicting aw, raw spectra produced slightly better models (*i.e.*, lower RMSEP), however, this was at the expense of a higher number of LV (3). Models developed on SNV and MSC data required only 1 LV to predict aw well (*R*^2^ > 0.97). Indeed, the difference in RMSEP between models developed on raw spectra and SNV or MSC pre-treated data was 0.01 or 2% of the range of aw values measured in wafer samples. Moisture content was best predicted for raw spectra for soybean (*R*^2^ > 0.95, 1 LV), while aw was best predicted with models using SNV and MSC (*R*^2^ > 0.91, 1 LV). The observation that spectral pre-treatments did not improve the prediction of MC for these samples indicates that some scattering information was informative for predicting MC. This is probably related to the swelling of the soybean during hydration. Conversely, the water activity prediction does not rely on scattering information since it is a measure of the free water in the system. With the exception of the coffee samples (for reasons explained above) applying scatter suppression pretreatments such as MSC and SNV generally improved model performance for prediction of aw while at the same time keeping low the number of LV required.

**Figure 3:**
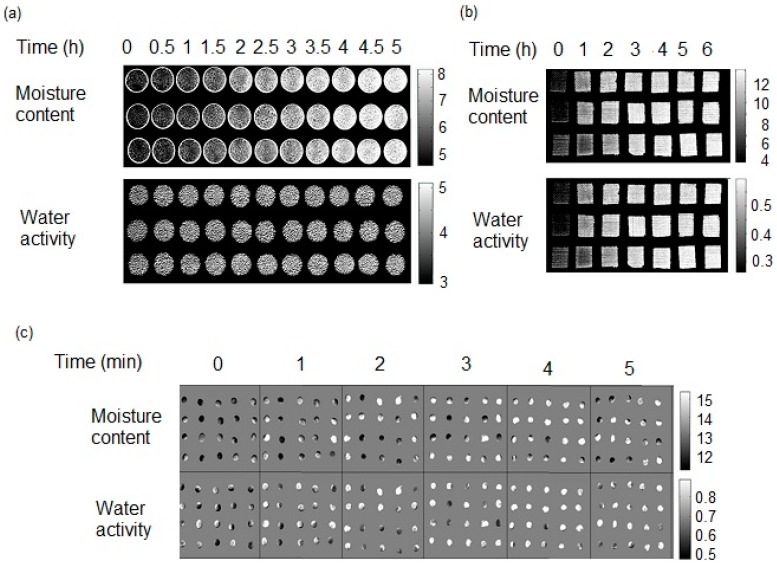
Predictions maps obtained by applying the best models for MC and aw to hyperspectral chemical images of (**a**) coffee (**b**) wafers and (**c**) soybean at different times. The scale bars at the side of each figure show the correspondence between pixel gray level and predicted moisture content or water activity.

Prediction maps for each product using optimally performing models are shown in Figure 3. It is possible to observe the variability in the distribution of MC and aw especially in soybean samples. These samples were soaked for short durations to change their moisture content and water activity, and for some samples this process caused the seed coat was crack. To overcome this variability in the samples when a simple spectrometer is used, it would be necessary to scan the same sample seed in multiple positions. This demonstrates the usefulness of HSI: Multiple samples can be imaged simultaneously. Prediction maps for coffee and wafer samples were more homogeneous, as would be expected for these more homogenous products. In the case of wafer samples, the prediction maps for MC and aw exhibited similar distribution at each sampling time. However, this was not the case for soybeans or coffee, indicating a larger deviation from linear behaviour for these products.

## 3. Experimental Section

### 3.1. Samples Employed

Low moisture systems used were: Instant granulated coffee, wafers and organic dry soybeans. Dry coffee and wafer samples were weighed and stored in sealed jars containing saturated solutions of NaCl for different times, and the weight gain of each sample was measured after specific time periods (0, 0.5, 1, 1.5, 2, 2.5, 3, 3.5, 4, 4.5 and 5 h for coffee and 0, 1, 2, 3, 4, 5 and 6 h for wafers). For soybean samples, 6 groups of 20 kernels were weighed and soaked in 10 ml water for different time periods (0, 1, 2, 3, 4 and 5 min), after which they were blotted dry using tissue paper and left to equilibrate for 10–15 min before measuring final weight. It should be noted that different samples were used at each time point. All samples, after final weight measurement, were imaged using the near infrared hyperspectral imaging system described below. Their water activity (aw) and moisture content (MC) was then measured using the procedures described below. Each experiment was repeated using new samples on 3 different days*.*

### 3.2. NIR Hyperspectral Imaging

Spectral images of each sample were acquired in the wavelength range of 880 to 1720 nm at 7 nm intervals using a line scanning near infrared hyperspectral imaging system (DV Optics, Padova, Italy). All data were stored in the ENVI format. The system consists mainly of an illumination source, diffuser, moving base, optics, spectrograph and camera. The moving base speed was set at 20 mm/s to obtain pixels of approximate size 0.3 × 0.3 mm. Before starting calibration and image acquisition, the system was turned on and allowed to stabilise for around 30 min.

The calibration procedure was as follows: 50 scan lines of a black reference (*I_b_*) were acquired and averaged by taking a measurement after covering the spectrograph lens with a cap; then a white tile with a known reflectance (*R_w_*) was placed on the moving base and used as a “white” reference (*I_w_*) by averaging 50 scan lines and finally the signal from the sample (*I_s_*) was converted and stored as reflectance (*R*) according to Equation (2):

(2) $$R=\frac{I_{s}-I_{b}}{I_{w}-I_{b}}R_{w}$$

### 3.3. Water Activity Measurement (aw)

For $a_{w}$ measurement, an AquaLab 3TE was used (Decagon Device Inc., Pullman, WA, USA). This dew point hygrometer was used according to the manufacturer’s specifications to measure the sample’s $a_{w}$ values. The $a_{w}$ meter was turned on and allowed to warm up for at least 30 min before measurement each day. The chamber temperature was set at 25 °C. Each sample of wafer and soybean was transferred to a sample cup and measured approximately 30 s after image acquisition to allow samples reach room temperature (25 ± 1 °C). Coffee samples were measured in the same cup used for image acquisition and was also allowed to equilibrate before awmeasurement.

### 3.4. Moisture Content Determination (MC)

Each sample’s moisture content was determined by oven-drying method as recommended for organic materials (AOAC, 1999) in specified time and temperature for each food sample in an oven (Gallenkamp Plus II, Sanyo Gallenkamp, Leicestershire, UK). Samples were weighed on an analytical balance (Sartorious Edgewood, NY, USA readability 0.0001). After weighing, the samples were transferred into the drying oven. For wafer and soybean samples, the temperature was set to 110 °C; samples were dried until constant weight was reached (difference <1 mg) while for coffee samples were 95 °C for 2 h. Thereafter, the samples were put in a desiccator filled with silica-gel for approximately half hour to cool down to room temperature, then the samples were weighed (w_d_), for determination of moisture content according to Equation (3):

(3) $$MC=\frac{w_{t}-w_{d}}{w_{t}}\;x\;100$$

### 3.5. Data Analysis

NIR hyperspectral images of hydrated soybeans (18 samples, 360 kernels), wafers (21 samples) and granulated instant coffee (33 samples) were imported from ENVI formatted files into Matlab v7.8 (The MathWorks, Inc., Natick, MA, USA). Data analysis was performed with in-house developed code and scripts. The spectra were trimmed to the range 950–1664 nm to remove the noisy part at both ends of the spectra. Data from 2 repetitions (*i.e.*, 2 different analysis days) were used to build the models (calibration set) and data from the other repetition was used to validate the models (validation set). In total, 22 coffee, 14 wafer and 12 soybean samples (240 kernels) were used for calibration and 7 wafer, 11 coffee and 6 soybean samples (120 kernels) were used for validation,. The average spectrum from each sample was used in model building. Partial least squares models were built to predict MC and aw, in the spectral range 950–1664 nm. The numbers of latent variables (LV) were selected by analysis of RMSECV (using leave one sample out cross validation) and jaggedness of the regression vector before made predictions for the validation set [14]. Improvement of model accuracy by means of spectra pretreatments was assessed; the pretreatments evaluated were: Standard normal variate (SNV), multiplicative scatter correction (MSC), first and second order derivative.

## 4. Conclusions

The results presented show that NIR hyperspectral imaging combined with chemometrics may be used to simultaneously predict water activity and moisture content in low moisture content systems and to visualise the spatial distribution of these attributes. In general the prediction models developed performed well (*R*^2^ > 0.9). However, as was shown for the coffee samples in this study, when a highly nonlinear relationship between the sample spectra and water activity exists, the proposed approach is less accurate. Further work is required to validate this technology in a commercial environment and develop robust multiband chemometric models suitable for online industry use.
